# Is there Progress? An Overview of Selecting Biomarker Candidates for Major Depressive Disorder

**DOI:** 10.3389/fpsyt.2016.00072

**Published:** 2016-04-25

**Authors:** Juan Joseph Young, Tim Silber, Davide Bruno, Isaac Robert Galatzer-Levy, Nunzio Pomara, Charles Raymond Marmar

**Affiliations:** ^1^Nathan Kline Institute, Orangeburg, NY, USA; ^2^Case Western Reserve University, Cleveland, OH, USA; ^3^MetroHealth Medical Center, Cleveland, OH, USA; ^4^Liverpool John Moores University, Liverpool, UK; ^5^New York University School of Medicine, New York, NY, USA; ^6^NYU Cohen Veterans Center, New York, NY, USA

**Keywords:** major depression, biomarker, multiplex, assay, diagnosis, trait, state

## Abstract

Major depressive disorder (MDD) contributes to a significant worldwide disease burden, expected to be second only to heart disease by 2050. However, accurate diagnosis has been a historical weakness in clinical psychiatry. As a result, there is a demand for diagnostic modalities with greater objectivity that could improve on current psychiatric practice that relies mainly on self-reporting of symptoms and clinical interviews. Over the past two decades, literature on a growing number of putative biomarkers for MDD increasingly suggests that MDD patients have significantly different biological profiles compared to healthy controls. However, difficulty in elucidating their exact relationships within depression pathology renders individual markers inconsistent diagnostic tools. Consequently, further biomarker research could potentially improve our understanding of MDD pathophysiology as well as aid in interpreting response to treatment, narrow differential diagnoses, and help refine current MDD criteria. Representative of this, multiplex assays using multiple sources of biomarkers are reported to be more accurate options in comparison to individual markers that exhibit lower specificity and sensitivity, and are more prone to confounding factors. In the future, more sophisticated multiplex assays may hold promise for use in screening and diagnosing depression and determining clinical severity as an advance over relying solely on current subjective diagnostic criteria. A pervasive limitation in existing research is heterogeneity inherent in MDD studies, which impacts the validity of biomarker data. Additionally, small sample sizes of most studies limit statistical power. Yet, as the RDoC project evolves to decrease these limitations, and stronger studies with more generalizable data are developed, significant advances in the next decade are expected to yield important information in the development of MDD biomarkers for use in clinical settings.

## Introduction

Major depressive disorder (MDD) is a highly prevalent illness in the United States that causes broad functional impairments (1) with significant public health costs (2, 3) and evidence of increasing rates over the past few decades (4). Together, this indicates that there is significant need to develop an objective characterization of the disorder for screening and diagnostics. The diagnosis of MDD currently relies on the clinical judgment of individual clinicians with high levels of subjectivity and potential variability. Following the publication of the fifth edition of the Diagnostic and Statistical Manual of Mental Disorders (DSM 5), concerns have been expressed with regards to the revised definition of MDD (5). Although based on opinion, the response to the changes of diagnostic criteria has highlighted how differing beliefs exist with regards to the MDD diagnosis, the subjectivity of diagnosing depressed patients, and the perception of a decrease in the reliability of MDD criteria under DSM 5 guidelines (5). Concerns about the validity of psychiatric diagnosis for depressive disorders is disconcerting and further emphasize the demand for more objective diagnostic modalities to assess MDD, such as blood-based and cerebrospinal fluid (CSF) biomarkers. Although there has been a significant amount of research in the development of fluid biomarkers for use in establishing MDD diagnosis (6–10), a consensus on which biomarkers are sensitive and specific enough to be used in a clinical setting has yet to be reached (11). In fact, studies of putative monoaminergic biomarkers such as peripheral and CSF levels of serotonin, dopamine, and noradrenaline often report conflicting results (12). Fortunately, there has also been an increased interest in other potential approaches by which MDD biomarkers may be discovered (13, 14). The objective of this article is to provide a broad overview of several types of biomarkers for MDD currently being investigated and to describe recent progress in identifying biomarkers that may potentially aid in the standardization of MDD diagnosis. Due to the sizeable literature investigating candidate MDD biomarkers and the limited space afforded to the authors, this overview will only focus on a select number of tissue-based biomarkers and recent multiplex studies published before December 1, 2015, while excluding current literature from the burgeoning neuroimaging biomarker data of structural imaging that has been previously reviewed (15–17).

## Biomarker Candidates

### Hypothalamic–Pituitary–Adrenal Axis (DST, DEX/CRH, Cortisol Response, Hypocretin)

HPA-axis hyperactivity has been associated with a spectrum of neuropsychiatric disorders due to its deleterious effects on the nervous system including dendritic process atrophy, decreased neurogenesis and neuroplasticity, and neuronal losses (18, 19); consequently, a wide range of biomarkers may be disrupted by HPA-axis dysfunction, such as disturbed adrenocorticotropic hormone (ACTH) regulation, dysfunctional corticosteroid receptor signaling, and glucocorticoid (GR) excess (18).

Furthermore, mutations in genetic regions involved in abnormal HPA-axis function (such as the FKBP5 allele) have also been associated with an increased risk for depression, and are similarly associated with abnormal plasma cortisol and ACTH concentrations (20–23).

However, beyond genetic factors, epidemiologic and clinical studies have determined that disturbances in HPA axis function have been consistently associated with biological changes in depression (24, 25). For example, one facet of depression history that is associated with HPA axis changes is early life stress. Early life stress (e.g., maltreatment or abuse) was found to result in HPA axis dysfunction during childhood and adolescence and contributed to an increased risk of developing MDD later in life (26).

Moreover, diminished cortisol suppression following dexamethasone (DEX) administration was observed in MDD patients with metabolic abnormalities of prefrontal and hippocampal regions, areas often related to MDD pathology (27). Other studies found that antidepressant treatment often resulted in decreased cortisol levels and a return to normal HPA axis function (28, 29).

Originally, as corticotrophin-releasing hormone (CRH) has been reported to be associated with increased depressive symptoms such as anhedonia and reduced appetite (30), a combined DEX/CRH test was thought to be capable of increasing diagnostic power over the Dexamethasone Suppression Test (DST) (12, 31). However, abnormal DEX/CRH results also occur in other psychiatric disorders resulting in lack of specificity as a diagnostic biomarker for major depression (12). Measuring cortisol levels is a more direct and accurate method of assessing HPA axis activity in depressed patients (32). Additionally, more recent studies focusing on cortisol measurements have demonstrated a link between cortisol levels and depression severity or depressive subtypes.

A recent meta-analysis (33) reports a significant association between HPA-axis hyperactivity as measured by elevated cortisol levels and the presence of melancholic or psychotic depression while lower cortisol levels were characteristic of depression with atypical features. For example, a longitudinal study of adolescents with depressive symptoms found that male adolescents with high morning salivary cortisol levels and increased depressive symptoms were more susceptible to the development of MDD demonstrating a sex-linked differentiation (34).

Another study also reported that persistent increases in cortisol awakening response (CAR) in adolescents more strongly correlated with higher levels of depressive symptoms than with anxiety symptoms (35). Lastly, a large cohort study confirmed increased CAR and dynamic cortisol secretion in depressed patients compared to controls in both current MDD and remitted MDD subjects, indicating that both measurements reflect an inherent risk in the development of depression (29). These studies suggest the use of morning salivary cortisol as a trait-like biomarker for developing preventative measures for high-risk populations, especially in asymptomatic individuals with possible genetic risks (36). However, a recent study revealed that increased CAR in healthy female adolescents significantly correlated with higher magnitudes of Profile of Mood States (POMS) subscale scores for “Tension-Anxiety,” “Depression-Dejection,” “Fatigue,” and “Confusion” (37) suggesting that morning salivary cortisol levels may also be descriptive of mood states and episodic depressive symptoms rather than characteristic of a purely trait marker for MDD. Such findings suggest variability in the use of salivary cortisol as a depression biomarker. However, it is important to consider how these contrasting conclusions may be affected by methodological heterogeneity and differences in subject populations among these studies.

Another possible biomarker source includes hypocretin, a neuropeptide that plays a role in sleep and arousal. Recently, it has been suggested that decreased numbers and size of hypocretin-containing neurons may be associated with the development of depressive symptoms including eating/drinking behaviors and disrupted sleep (38, 39). One study found that hypocretin levels in the CSF of MDD-diagnosed patients with high suicidal ideation were significantly lower than those of patients with dysthymia and adjustment disorder (40). Additionally, hypocretin levels correlated significantly with CSF levels of other peptides that affect sleep and appetite including delta sleep-inducing-peptide-like immunoreactivity (DSIP-IL), CRF, and somatostatin. Not only are these results indicative of the diagnostic utility of measuring hypocretin concentrations but also these peptides may also be useful in discriminating affective disorders by associating differing biological characteristics with signs and symptoms of depression. However, one study reported results that counter the more common conception of lower hypocretin levels in depression (41). Bearing in mind that the relatively few studies and the dynamic character of HPA axis components in general concerning hypocretin-based biomarkers for depression, future studies would be instrumental in further elucidating hypocretin effects in depressed patients.

### Thyroid Function and Thyroid Autoimmunity

A number of studies have related thyroid dysfunction with depressive symptoms and depressive disorders (42–50). However, a direct correlation is indeterminate as evidenced by a number of conflicting studies (51–56). More recent studies have shown a relationship between levels of antithyroid antibodies with depression (57–59) and poorer “psychosocial well-being” (60). However, there also exists literature demonstrating equivocal data concerning this association (61). In fact, one group found that thyroid function and thyroid autoantibody levels were not associated with depression severity despite an association with the presence of depressive symptoms (58). Supporting these results, a general population study showed no significant difference in depressive symptoms between euthyroid individuals and those characterized to have subclinical hypothyroidism (62). Conversely, another general population study found an increase in prevalence of lifetime depression diagnosis in subjects positive for thyroid peroxidase antibodies, suggesting its use as a trait marker for depression despite finding no association between depression disorder diagnosis and TSH or free T4 levels (63). Interestingly, one study (64) found T3 and T4 levels derived from hair were significantly lower in patients concurrently having a depressed episode (*P* < 0.001), which may indicate the use of thyroid hormones as a state-like biomarker. In this sense, future studies should focus on readily accessible markers of thyroid function that have some state-like diagnostic utility in major depression diagnosis as studies researching their use as trait-like markers have demonstrated mostly equivocal results.

### Cytokines and Inflammatory Markers

There also exists an abundance of evidence that elevated proinflammatory cytokine concentrations and an increased immune response are associated with depression diagnosis, symptomatology, and severity (65–69). Reflective of this significant proinflammatory response in depressive disorders, a recent proteomic study found elevated levels of acute phase reactants (e.g., ferritin, serotransferrin, Haptoglobin-related protein, ceruloplasmin) and proinflammatory markers (e.g., IL-16, MIF, Tenascin-C, and EN-RAGE) in drug-naive MDD patients, indicating a disorder-related increase in immune processes (70). These findings are supported by neuroimaging and animal studies, which have demonstrated that alterations in neuroplasticity promote manifestations of depressive phenotypes as a result of cytokine-induced neural apoptosis and metabolic dysregulation (71). Further, inflammatory cytokines have been described to alter basal ganglia processes leading to common depressive phenotypical characteristics including anhedonia, fatigue, and psychomotor retardation (72–75). A recent review also reported increased neopterin levels in a number of studies of depressed patients, specifically in melancholic subtypes of depression (76). Additionally, inflammatory markers IL-6 and soluble intercellular adhesion molecule (sICAM) have been associated with sleep disturbances in depressed patients (77). IL-8 and TNF-α have also been reported to remain elevated in certain subsets of depressed patients after antidepressant therapy, indicating possible trait characteristics (78).

C-reactive protein (CRP) and interleukin (IL)-6, specifically, have been found to exhibit trait characteristics (i.e., gender effects, impact of early life adverse events) as an inflammatory biomarker for depressive pathology (79–82). In their meta-analysis, Valkanova and colleagues (83) found that these two putative analytes had a small but significant association with the development of depressive symptoms, indicating the presence of raised inflammatory markers preceding the development of MDD. However, the authors cautioned that their results might have limited significance due to heterogeneity (i.e., of depression, methodologies, populations, etc.) across studies.

For instance, one study (84) found both higher and lower levels of different inflammatory markers in major depressed patients depending on the presence or absence of melancholic features, indicating that that the overall characteristics of depressive symptoms were more associated with the composition of inflammatory profiles and less so on concentrations of individual markers. Moreover, a study of an elderly population found that when controlling for age-related chronic diseases, CRP was not a statistically significant marker associated with the presence of MDD or sub-threshold depression (85). Lastly, one group reported significantly lower levels of IL-6 in subjects with high self-reported depressive symptoms while showing no significant differences of IL-8, IL-10, and TNF-α levels when compared to controls (86). As a whole, these studies demonstrate the complexities of relying on individual inflammatory marker concentrations to characterize generalized depression.

Yet, there is reasonable evidence that suggests inflammatory responses are more prominent in certain subsets of MDD than others. A study evaluating biomarker associations with depressive subtypes found that increased inflammatory markers (i.e., CRP, IL-6, TNF-α) were significantly associated with atypical depression as compared to typical or melancholic depression (87). Consistent with these results, a more recent study (88) reported that elevated IL-6 levels were consistently higher in patients with atypical depression. Similarly, a recent study has detected consistently increased CRP levels in depressed patients with comorbid diabetes mellitus (89). Moreover, these studies reinforce clinical evidence that both inflammatory diseases and depression are often associated with comorbid illnesses like metabolic disorders (87, 90), especially in more elderly subjects (91–93). It is possible that in many cases, the predisposition to depression in patients with elevated inflammatory biomarker concentrations is affected by a number of outlying factors often present before the emergence of the first depressive symptoms. Therefore, in addition to their lack of specificity to MDD, the diagnostic value of individual inflammatory biomarkers could be hindered by some inherent heterogeneity of depression. Although they may be useful as MDD biomarkers in a research environment, their low sensitivity and specificity (6) prevent them from being utilized in the majority of clinical settings.

In contrast to the relatively limiting findings of individual inflammatory analyte concentrations as biomarkers for depression, inflammatory markers have potential use as state markers by characterizing treatment response to antidepressants (94, 95). Significantly, antidepressant effects are associated with a decrease in proinflammatory/anti-inflammatory protein ratios, especially in patients that respond to treatment when compared to non-responders or healthy controls (96). Specified inflammatory markers tend to correlate well with treatment efficacy in depressed patients. For instance, TNF-α levels have been reported as a marker of treatment response and psychopathological improvement (94, 97). However, a recent meta-analysis (98) failed to detect pharmacological effects on serum levels of TNF-α, although they reported that IL-1β levels decreased after antidepressant treatment. Another analyte, high sensitivity C-reactive protein (hs-CRP), was found to be a highly specific baseline biomarker when evaluating patient response to Infliximab in treatment-resistant depression (TRD) (99). Similarly, CRP levels have been used to differentially evaluate treatment efficacy between escitalopram and nortriptyline (100). Considering the high incidence of treatment resistance in MDD-diagnosed patients, inflammatory markers capable of determining antidepressant treatment response will have a significant impact on depression management and allow practitioners the ability to modify treatment plans according to personalized histories and peripheral biomarker results. For further review, please read the following articles: Dantzer et al. (101), Leonard and Maes (102), Miller et al. (9, 75), Müller and Schwarz (103), Raison and Miller (104), and Young et al. (105).

### Markers of Oxidative Stress

Oxidative stress has also been proposed to have an important role in depression pathology (102, 106–108). Consistent with preclinical studies that display increased antioxidant capacity with antidepressant therapy (109–111), human studies have demonstrated that increased oxidative activity is reversible by SSRI action in severely depressed, medication-naive patients (112) or melancholic patients (113), implying the involvement of oxidative processes in depressive disorders and monoamine metabolism. However, one study (114) found that treatment with antidepressants did not affect oxidative–antioxidative markers in MDD subjects while another found increased oxidative stress after treatment (115). An explanation for these inconsistent results may be the varying oxidative effects of different antidepressant formulations and duration of treatment that vary between studies. Whatever the case, the extensive literature associating oxidative processes and depression suggests markers of oxidative stress may be able to identify depressed patients and quantify severity.

Several studies have found significantly increased oxidative stress markers [e.g., 8-hydroxy-deoxyguanosine (8-OHdG), F2 isoprostane, peroxidase, malondialdehyde (MDA), and superoxide dismutase (SOD)] and decreased antioxidative capacity in MDD patients (112–118). Some studies have also demonstrated specific correlations of depressive subtypes or features with oxidative stress, yet results remain conflicting. Decreased GSH has also been found to correlate with severity of anhedonia in depressed patients (119), while plasma GSH-R and erythrocyte glutathione peroxidase (GPX) levels were elevated in MDD patients with melancholic features (113). A study determining the relationship between psychological responses and 8-OHdG levels found a positive correlation with depression–rejection scores of the POMS scale in females compared to a negative correlation in men, suggesting gender differences in depression-associated oxidative damage markers (120). Additionally, increased expression and distribution of allele frequencies of enzymatic proteins involved in the production of oxidative free radicals (e.g., inducible nitric oxide synthase and myeloperoxidase) were characteristic of patients with recurrent depression (121). Recently, Smaga and colleagues (122) completed a review of a number of clinical studies that demonstrated higher oxidant status in depressed patients including higher plasma peroxide levels, higher nitric oxide levels in serum, and higher xanthine oxidase levels. The authors also found that oxidative DNA damage and higher levels of lipid peroxidation markers were also prevalent in a number of depression studies.

In elderly populations, free radicals have been implicated in the pathophysiology of other neurodegenerative disorders along with MDD (123, 124). A study by our group has shown increased CSF F2-isoprostanes in geriatric patients diagnosed with MDD; further, an inverse relationship was found between amyloid-β 42 and F2-isoprostane CSF levels, suggesting that increased oxidative stress pathology may be associated with increased brain amyloid burden (125). These findings are corroborated by other published findings that imply similar pathological mechanisms (e.g., increased levels of lipid peroxidation) between MDD and chronic, age-related diseases (126). Due to the complex neurobiological complications that are present in late-life depression, there is a need to identify specific markers in order to direct biology-based treatment.

### Neurotrophins

Neurotrophins [i.e., nerve growth factor (NGF), brain-derived neurotrophic factor (BDNF), neurotrophin (NT)-3, -4, and -5] are homodimeric proteins that interact with the tropomyosin receptor kinase (Trk) family of receptors through which they mediate the processes of neurogenesis and neural plasticity in both the peripheral and central nervous systems (127). Several connectomic studies have increasingly indicated the disruption of integral whole-brain structural networks in MDD, suggesting the presence of abnormal neuronal synapse formation within certain populations of depressed patients (128, 129). In fact, there is evidence that disrupted neurogenesis may be a characteristic of MDD pathophysiology, especially of the hippocampus (130, 131). Due to the role of NTs in neuroplastiticty, their use as potential biomarkers has often been reviewed. Of these, the most researched is BDNF, with studies finding its downregulation in the limbic structures of chronic-stress exposed rats and reports of decreased peripheral levels in MDD patients (132–136). Significantly, a recent study (137) has shown serum BDNF may also have significant potential as a discriminatory diagnostic tool for first major depressive episode (MDE) patients, prompting the need for more expansive studies concerning its use in clinical settings. While there have been conflicting results concerning correlations of depression severity with BDNF levels (138), BDNF concentrations have been reported to increase after antidepressant therapy with more prominent elevations in patients with higher baseline depression severity (139–145). Several studies have also reported elevated BDNF levels in responders to antidepressant treatment compared to non-responders that continued to demonstrate lower BDNF concentrations after pharmacologic management (146, 147). These findings suggest that BDNF may be utilized as a state marker to assess psychopharmacological therapy and prognosis of individual MDD patients (148), although the effect on BDNF levels may vary between different classes of antidepressants (149). Ultimately, due to its intrinsic function in influencing the development and maintenance of a patient’s cognitive abilities, BDNF could have potential for evaluating other therapy effects involving learning, memory, and executive functions (134, 150).

Alternatively, de Azevedo Cardoso and colleagues (151) have suggested that BDNF may also have trait-like properties. For example, differences between male and female BDNF levels have been associated with contrasting antidepressant effects between the two genders (145). Additionally, there is evidence that BDNF levels are more negatively affected in patients with chronic depression who have experienced more adverse life events (152). Supporting this theory, several groups have illustrated how BDNF genotypic variations were associated with risk for depression (151, 153–157). BDNF DNA methylation patterns have also been associated with depression severity, and the presence of suicidal ideation in MDD subjects (158–160). Consequently, the potential for BDNF to be a trait and state-like marker makes it one of the more versatile biomarker candidates being researched today.

In contrast, fewer studies have focused on other NTs, with the notable exception of NGF, which has been found to be increased during circumstances that cause anxiety or anticipation of anxiety (161). Regarding NGF’s relationship to depressive symptoms, reports of NGF concentrations have yielded conflicting results (151, 162, 163). Additionally, due to its association with other affective disorders such as bipolar disorder (BPD) (164); it is unlikely to be specific to MDD. These limitations, however, should not preclude it from further research.

### Markers in Genetics and Genomics for MDD

Past studies have suggested that there is a complex genetic component to the development of MDD, with evidence that heritability is a key factor in a significant number of depression cases (165–167). Additionally, several studies have revealed various polymorphisms and overexpression of certain genes in patients presenting with depressive symptoms (168–170). One example is a blood-based study that found increased serotonin type 1A receptor (5-HT_1A_) expression within platelets of MDD patients compared to controls (171). The authors also reported decreased levels of serotonin (5-HT), platelet poor plasma (PPP) 5-HT, and a decrease of the 5-HT metabolite, 5-hydroxyindoleacetic acid (5-HIAA), suggesting that increased 5-HT_1A_ expression inversely correlated with 5-HT activity *via* a negative feedback mechanism. Often, such genetic variants imply pathological mechanisms associated with the dysfunction of different biological systems implicated in depression. Another example is HPA axis hyperactivity, which is believed to influence the pathogenesis of MDD due to findings of GR and mineralocorticoid (MR) receptor dysfunction in depressed patients (24). For instance, a longitudinal study focusing on neuropsychiatric disorders in an elderly community found that several single-nucleotide polymorphisms (SNPs) of angiotensin-converting enzyme (ACE) were significantly associated with the risk of late-life depression (172). Additionally, they reported that two SNPs (rs4291 and rs4295) were associated with the risk of incident depression over the study’s 10-year follow-up. More recent studies have determined that polymorphisms of the FKBP5 gene (a gene that plays a role in immune regulation) also modulate GRs, and have been associated with the development of depression (20–23). A meta-analysis of HPA axis dysfunction associated with GR abnormalities found that glucocorticoid-induced leucine zipper (GILZ), a product of GR-initiated gene transcription, has been suggested to be associated with biological pathways relevant to depression (173). Though few studies have focused on GILZ concerning depressive disorders, there is clinical evidence that a reduction in its expression is associated with reduced hippocampal volumes found in MDD-diagnosed subjects (174).

More comprehensive data concerning the heritability of depressive disorders will likely come from the increasingly complex genome-wide research being conducted today. For example, the GeneQol Consortium (175) gathered data from a substantial number of studies that undoubtedly demonstrate the involvement of genetic variables in quality of life (QOL) domains (e.g., fatigue, pain, general functioning, social functioning, general health). Of the biomarker candidates they reviewed, candidate genes and molecular markers that had the most evidence of association with QOL domains were genes for inflammatory cytokines (e.g., IL-1β, IL-6, IL-8, and TNF-α). Additionally, inflammatory markers (e.g., CRP) and anti-inflammatory markers (e.g., IL-1RN, IL-1RA, and IL-10) were also associated with a smaller number of QOL domains. Other QOL-associated markers include genes for dopaminergic and serotonergic synapses [MAOA, 5-HTT (SLC6A4), TPH1], the glutathione metabolic pathway (DPYD), and pain receptor pathways (OPRM1). However, the specificity and accuracy of these markers for MDD may be limited by significant genetic heritability among psychiatric disorders (176), and the fact that current MDD genomic data is limited by heterogeneity and insufficient power (177). Yet, these findings still underlie the potential of genetic irregularities to play a role in more accurately characterizing and diagnosing depressive disorders. Currently, better powered studies are required to determine the etiologic and genetic variables involved in MDD pathology, especially when conducting genome-wide research.

MicroRNAs (miRNAs) are a popular genetic marker in researching MDD biomarkers due to their role as small RNA regulators involved in neural stem cell proliferation, neurogenesis, and neural plasticity (178). In addition, several miRNA alterations were associated with an increase in risk for major depression and negatively regulate the expression of either serotonin receptors (SERT) or 5-HT_1B_ receptors (179). Significantly, one study (180) has indicated that miRNA profiles are capable of separating MDE patients from controls while a second study (181) found 30 miRNAs to be differentially expressed in MDD patients after escitalopram treatment. These findings are further corroborated by results from a study demonstrating gene variations in Drosha RNase and Digeorge syndrome critical region 8 (DGCR8), a known cofactor in miRNA processing, and AGO1, a component protein involved in the production of mature miRNAs, as being capable of significantly differentiating MDD patients and healthy controls in relation to genotype and allele frequencies (182). Another study demonstrated that dysregulation of circadian rhythms in MDD patients was associated with the rs76481776 polymorphism of miR-182, suggesting that symptoms of MDD may be inherently linked to genetic variations that affect miRNA function (183). These distinctive miRNA profiles in depressive disorders predispose them to becoming a promising source of biomarkers for MDD research and diagnostics. With more studies confirming their involvement in depression and with advances in miRNA expression measurement techniques (184), miRNA data may prove to be useful additions to MDD biomarker panels.

Several studies have demonstrated an association between telomere length and depressive disorders. Szebeni and colleagues’ recent post-mortem study, previously described, found decreased expression of telomerase reverse transcriptase (TERT), an enzyme whose function is to prevent telomere shortening (TS), in oligodendrocytes derived from different parts of the brain (185). Another study found over expression of certain genes involved in propagating TS in the leukocytes of female MDD subjects (186). Specifically, these genes have been associated directly or indirectly with telomere dysfunction (STMN1, P16^ink4a^), oxidative stress (OGG1), and aging (OGG1) while others (FOS, DUSP1) were linked to the stress-related p38MAPK pathway, although they are not specific to depression and may be found in normal aging or anxiety disorders (186). In fact, at least one large-scale study has shown an association between symptoms of anxiety and TS in comparison to depression-associated telomere dysfunction over a 2-year period of time (187). Considering that telomere length is a biomarker of cellular aging, it is not surprising that it is more commonly associated with chronic periods of life-long depression rather than acute episodes (188).

Yet, shorter telomere lengths have also been observed in children of lower socioeconomic status with coexisting dopaminergic/serotonergic genetic sensitivity to harsher social environments (189). This study suggests that significant stress at an early age may be associated with genetic and biological changes that predispose children to depressive disorders. Therefore, TS may not be exclusively valuable as a biomarker in older populations, but may also be useful in identifying children who are more prone to TS as a result of immature protective mechanisms against inflammatory, oxidative, and HPA-axis effects on cellular genetic coding. Furthermore, a recent study reported a negative correlation between telomere length and cortisol reactivity in female adolescent subjects with familial risk for depression (190). This study implies how inherent HPA axis dysregulation, consistent with biological changes in depression pathology, is associated with TS that typifies the accelerated cellular aging in younger cohorts (190). Accordingly, such studies indicate TS could find more use as a predictive or screening marker in younger and geriatric populations, respectively, than a specific biomarker for MDD. However, a recent large-scale study found increased mitochondrial DNA and shortened telomere length in subjects with major depression status, but did not find either variable to correlate with increased risk of developing major depression, suggesting characteristics of a state biomarker (191). Further research will be required to elucidate the basis for these contrasting findings.

Lastly, significant consideration should be given to the difficulty of directly associating genetic phenotypes with psychiatric disorders. In response to this challenge, Gottesman and Gould (192) proposed criteria for developing endophenotypes, intermediary constructs that would act as tractable traits that could more effectively characterize the heritability of psychiatric disorders. Hasler et al. (193), and more recently, Goldstein and Klein (194) have published detailed reviews about both psychopathological (e.g., neuroticism, anhedonia, depressed mood, increased stress sensitivity), and biological (e.g., morning cortisol, tryptophan depletion, DEX/CRH, CRH dysfunction, hippocampal volume, and reduced 5HT1A receptor expression) endophenotypes for depression. However, there continues to be a relative lack of evidence for current putative endophenotypes, specifically due to a deficiency of family and twin studies (194). It is therefore possible that future endophenotype studies and analysis may contribute to the growing literature characterizing MDD as well as further the development and understanding of MDD etiology and pathophysiology that remain the most heterogeneous components of the disorder.

### Epigenetics

Epigenetic mechanisms have been used to explain how early life exposures to toxic or stressful stimuli may contribute to the predisposition or development of mental illness (195). For depressive disorders, histone modification at the amino (N)-terminal tails and DNA methylation has been the most studied in determining how epigenetic factors affect the progression, severity, symptomatology, and treatment response of depression (196, 197). Significantly, these epigenetic modifications may affect expression of certain receptors (e.g., GR receptors in the hippocampus), which leads to either an increased or decreased risk for depression in the future (196). This is supported by animal studies that show antidepressant-like effects of histone deacetylase inhibitors (195, 196, 198–200), which are thought to induce histone acetylation in certain regions of the brain. Overexpression of DNA methyltransferases also leads to an increase in DNA methylation and has been associated with abnormal dendritic spine plasticity and alterations in behavioral responses. Supporting this, one group found site-specific hypermethylation of TrkB-T1 to be increased in suicide completers (201), suggesting a pattern of methylation abnormalities in subjects with depressive phenotypes. Recently, epigenome-wide association studies have demonstrated several genes with methylation associations in depressed subjects compared to controls. As these studies are mostly array-based, they have had the advantage of investigating the entire genome, but replication studies are currently lacking (202). One recent genome-wide study (203) was able to separate medication naive MDD subjects from controls by observing differences at 363 CpG sites that differed from the pattern they observed in their schizophrenia patients (204) indicating disease-specific patterns. Furthermore, several candidate gene studies involving DNA methylation have been investigated and include genes that have been previously implicated in depression. Among these genes, SLC6A4, BDNF, and NR3C1 have been the most studied, with BDNF methylation having the most consistent data concerning associations between DNA methylation and depressive symptoms/antidepressant response (202). This study demonstrated a significant association between depression and methylation levels of BDNF at specific CpG sites. Notably, the authors have shown that such robust biomarkers may come from easily obtainable specimens such as buccal samples. Although epigenetic research is still in its infancy, these epigenetic mechanisms and resulting patterns in chromatin remodeling are becoming established as a basis by which chronic social defeat, early life stress, variability of maternal care, and antidepressant therapy may influence the progression or resolution of depressive symptoms (197, 202, 205, 206). Further studies and elaboration on these mechanisms will likely lead to significant advances in the development of an epigenetic model from which MDD biomarkers may be retrieved. Please see Nestler et al. (195), Tsankova et al. (197), and Januar et al. (202) for further review.

## Proteomics, Metabolomics, and the Utility of Multiplex Assays

### Proteomic and Metabolomics Research

There have been recent technological advances that have allowed more in-depth characterization of medical disorders on both the analytical and clinical level. Mass spectrometry (MS) proteomics has allowed researchers to quantify expression levels of proteins for detecting changes after translation or protein interactions (207). High performance liquid chromatography (HPLC) has been used to separate and assess proteomes/metabolites in both schizophrenia (208) and BPD (209). With depression, Martins-de-Souza’s group was able to observe differing levels of various proteins involved in metabolic pathways and molecule transport between MDD subjects and control subjects (*P* < 0.05) (210). Interestingly, they found that those with MDD who developed psychosis had differentially expressed proteins that were different from MDD subjects who did not develop psychosis. Thus, their report suggests that proteomes may aid in the characterization of MDD subtypes and the varied symptomology of psychiatric patients. There has also been an increase in use of high-resolution nuclear magnetic resonance (NMR) spectroscopy to evaluate biofluids to not only document baseline levels of metabolites but also produce complete time-lines of metabolite variability that may result from drug administration or medical disorders (211). Consequently, a number of recent studies have taken advantage of these more complex analytical tools to search for possible MDD biomarkers in different biological systems.

Using gas chromatography/MS (GC/MS) coupled with multivariate statistical analysis, Ding and colleagues were able to produce distinct blood-based metabolic profiles that were able to separate MDD patients from healthy controls (212). Critically, their study found significant separation between a subgroup of MDD patients with “early life stress” (ELS) versus those that did not have ELS, indicating possible use for characterizing depressive subtypes. Their investigation further supports the theory of separate pathophysiologic mechanisms that cause differing metabolite concentrations between MDD subtypes. This is a significant finding given that ELS has been considered a preventable risk factor for a number of pathological psychiatric disorders (213). Similarly, Zheng and colleagues (214) used a GS/MS-based urinary metabolite signature to demonstrate significant separation of MDD from controls in both training samples and an independent test cohort that included medicated MDD subjects. Another group used HPLC to evaluate plasma levels of glutamic acid, aspartic acid, glycine, gamma-aminobutyric acid (GABA), and nitric oxide (NO) of medication-naive melancholic MDD patients, and differentiate them from matched controls (215). The resulting data indicated that plasma GABA levels were associated with anhedonia and suicidal ideation in affected MDD subjects. The authors observed that the studied analytes could be used as trait-like biomarkers since metabolite plasma concentrations continued to be abnormal even after 2 months of fluoxetine treatment despite having no significant correlation with Hamilton Depression Rating Scale (HAM-D) scores or severity of depression. The results of this study may indicate how dysregulation of the metabolism of monamine neurotransmitters may vary and predict the course of depression in certain individuals. Ditzen et al. (216) used 2D polyacrylamide gel electrophoresis and time-of-flight MS peptide profiling to determine differences in CSF proteomes between depressed patients and controls finding 11 significantly differentially expressed proteins and 16 phosphorylated proteins that separated the two groups. These proteins have been implicated in CNS diseases, nervous system development, and cell death. Additionally, Stelzhammer and colleagues (70) have demonstrated a number of proteomic changes in first onset, drug-naive MDD patients including markers of inflammation (ferritin, EN-RAGE, ceruloplasmin, IL-16, serotransferrin, tenascine-C), oxidative stress (cortisol), RAS markers (ACE), and changes in growth factors (BDNF and GH). Lastly, Wang and colleagues (217) have also reported consistently high sensitivity, specificity, and accuracy in discriminating between MDD subjects and healthy controls by using matrix-assisted laser desorption ionization time-of-flight MS to determine peptide profiles in first episode, drug-naive MDD. The potential for a laboratory-based analysis to aid in MDD patient identification validates future research using these developing technologies along with further evaluating any candidate biomarkers found to be capable of discriminating affective disorders.

### Emerging Multiplex-Based Biomarkers

Though there have been a number of studies analyzing the various neurobiological features persistently found in depressed patients, no specific marker from a single biological system has been capable of significantly improving upon the current diagnostic criteria set for MDD patients. As several of the aforementioned biomarkers seem necessary but not individually sufficient, multiplex assays are currently the most promising to contribute consistent results to aid in further standardizing MDD diagnosis and research. As past studies have demonstrated, depression pathology is influenced by disruption from multiple systems including the HPA axis, oxidative pathways, inflammatory processes, and neurotrophic homeostasis. Collectively measuring the putative analytes of each system will likely increase the power of any diagnostic panel developed for MDD. This concept is supported by studies that used multiple analytes of different origins and considered to be potential biological markers of depressive disorders to increase specificity and sensitivity in diagnosing MDD. One study worth noting achieved high sensitivity (above 90%) and specificity (above 80%) in distinguishing MDD patients from healthy controls (218). The authors used nine biomarkers from different biological sources such as inflammatory and oxidative indices (α1 antitrypsin, apoplipoprotein CIII, myeloperoxidase, soluble TNFα receptor type II); the HPA axis (epidermal GF, cortisol); neurogenesis (BDNF); and metabolism (prolactin, resistin) to develop an algorithm that produces a score that could potentially be used for an objective diagnosis of MDD. In addition to achieving high sensitivity and specificity in their pilot study, Papakostas et al. also produced a similar performance in their replication study. This group further refined their model algorithm by factoring in gender, BMI, and normalized cortisol levels (219). Another group used CSF concentrations of multiple analytes including inflammatory biomarkers (IL-6), serotonin metabolites (5-hydroxyindoleacetic acid), dopamine metabolites (homovanillic acid), and HPA axis biomarkers (hypocretin) to detect severe suicidal behavior and increased risk of completing suicidal attempts in MDD patients (220). Likewise, CSF protein biosignatures were found to be capable of discriminating depressed, bipolar, and schizophrenic patients from healthy controls (221). These markers included proteins involved in neurogenesis (e.g., neuronal growth regulator 1, neural proliferation differentiation and control protein); neurotransmission (seizure-related 6 homolog protein); and oxidative damage (GPX 3). However, Maccarrone and colleagues have indicated difficulties differentiating between individual psychiatric disorders and controls, as only a few proteins of their CSF biosignatures were found competent enough to distinguish between disease groups. They have reported high accuracy rates of distinguishing bipolar, depressed, and schizophrenic patients (i.e., 83.3% for MDD). Other multiplex studies that have been discussed in previous sections have also shown significant inflammatory/oxidative features (70) and epigenetic variations (203) in MDD subjects. Due to their inherent sophistication and more comprehensive analysis relative to individual markers, these multiplex assays have the potential to reduce inconsistent data that develop due to differences in study populations and methods seen in past, single biomarker studies (219). However, it is currently imperative to conduct future studies that focus on replicating and confirming such findings that yield increased MDD diagnostic accuracy using these methods.

## Limitations of Current Research

The main variables that are consistently problematic in the development of a reliably viable MDD biomarker involves the heterogeneity of depressive disorder pathophysiology, etiology, and study designs, which in turn may contribute to conflicting data. As a result, variations between studies reviewed here limit the precision and generalizability of the findings. Additionally, although with notable exceptions mentioned [e.g., the ADNI study and Vreeburg et al. study (29)], most studies we reviewed collected data from small samples sizes often consisting of fewer than 100 subjects. Another difficulty is how to consistently associate biomarkers with DSM criteria for MDD (e.g., low mood, poor concentration, suicidal ideation), which are not always necessary in diagnosing depression and could be present in other psychiatric disorders including schizophrenia. Consequently, any biomarkers that are heavily associated with non-specific clinical symptoms of depression may produce a high rate of false positives. This is significant as the majority of studies focus on exploring biological differences between depressive disorders and control groups, but do not extensively evaluate putative biomarkers’ diagnostic specificity against other psychiatric disorders. Although current research has an increasing neuroscience focus advocated by the National Institute of Mental Health through the novel Research Domain Criteria (RDoC) project (222), we are likely decades away from discovering the basic underpinnings of neurobiological changes present in psychiatric disorders and how they relate to behavioral shifts; discoveries that are necessary to determine the adequacy of developing biomarkers (223). Consequently, the only standards available to compare the validity and specificity of diagnostic biomarkers are syndromic and descriptive categories developed by expert consensus (224). Although the most recent research on MDD biomarkers has suggested the possibility of finding more objective forms of diagnostics compared to the aforementioned diagnostic criteria in clinical use today, it is still unclear how these discrete markers would relate to the diverse clinical presentations and differing populations that continuously confound research on MDD.

## Conclusion

Multiple biological pathways are robust sources of tissue-based MDD biomarkers with trait and state characteristics. However, individual biomarkers currently impart limited clinical utility. In the future, multiplex assays comprised of putative depression biomarkers may improve upon the clinical evaluation of MDD, assess treatment efficacy, and serve to standardize discharge criteria. However, independent replication studies with large sample sizes are needed to fully substantiate the validity of such panels. Furthermore, the use of these markers are limited by high costs and confounding factors associated with each component of prospective diagnostic constituents (225). If these markers become reproducible and translate into readily available diagnostic tools with ease of access, low cost, rapid formulation, and high sensitivity/specificity, the implications for clinical use would be tremendous. After decades of investigations and several promising markers falling into obscurity, it is difficult to say whether we are getting closer or farther away from one of the holy grails of diagnostic biomarkers for depression. Suffice it to say, every study that contributes to the development of such biomarkers will assuredly be needed if such a goal is to be achieved. As the RDoC project and current technology evolve to lessen the limitations of past studies, future large-scale MDD biomarker studies will be necessary to yield advances that will hopefully have utility in the clinical setting.

## Author Contributions

JY, TS, DB, IG-L, NP, and CM contributed to the conception and design of this manuscript, revised the manuscript after critically appraising all information contained in the manuscript, and approved the final version.

## Conflict of Interest Statement

The authors declare that the research was conducted in the absence of any commercial or financial relationships that could be construed as a potential conflict of interest.
