# Supplementary material for: Is there Progress? An Overview of Selecting Biomarker Candidates for Major Depressive Disorder
Source: Front Psychiatry. 2016 Apr 25;7:72. doi: 10.3389/fpsyt.2016.00072 (PMC4843170; doi:10.3389/fpsyt.2016.00072)
Supplement: Supplementary file 1 [file Table_1.DOCX]

Supplementary Material

**Is there Progress? An Overview of Select Biomarker Candidates for Major Depressive Disorder**

Juan Joseph Young, MD, Tim Silber, Davide Bruno, PhD, Isaac Galatzer-Levy, PhD, Nunzio Pomara, MD, Charles Marmar, MD

**Correspondence:** Charles R. Marmar, M.D, Department of Psychiatry, New York University (NYU), Langone Medical Center. 1 Park Avenue, 8th Floor New York, NY 10016 United States. Charles.Marmar@nyumc.org

# Supplementary Table

| Table 1. Summary of Select MDD Biomarker Studies | | | | |  | |  | |
| --- | --- | --- | --- | --- | --- | --- | --- | --- |
|  | |  |  |  | |  | |  |
| Biological  System or Study Focus | | Study | Biomarker Candidate(s) | Sample | | Changes in Depression* | |  |
|  | |  |  |  | |  | |  |
|  | |  |  |  | |  | |  |
|  | |  |  |  | |  | |  |
| HPA Axis | | Alvarez *et al.,* 2013 | Urinary Cortisol | N=61 (21 MDD - 57.1% F, mean age 63y) | | ↑ free urine cortisol in patients with both DM and MDD compared to those without MDD (p=0.03) | |  |
|  |  |  |  |  | |  | |  |
|  |  | Brundin *et al.,* 2007 | CSF Orexin | N=66 (32 with MDD [53% F; mean age 44y], 23 with dysthymia [64% F, mean age 40y], 11 with adjustment disorder [48% F, mean age 33y]) | | ↓ Orexin in CSF of MDD vs. dysthymia and adjustment disorder patients (one-way ANOVA, p=0.001; Bonferoni-Dunn’s post hoc-test p<0.01) | |  |
|  |  |  |  |  | |  | |  |
|  |  | Nelemans *et al.,* 2014; | Salivary Cortisol | N=184 adolescents (43% F, mean age 14.99y) | | ↑ cortisol awakening response:  high CAR group had higher depressive symptoms vs. low CAR group (p=0.01) | |  |
|  |  |  |  |  | |  | |  |
|  |  | Owens *et al.,* 2014; | Salivary Cortisol | N=1,858 adolescents divided into Cohort 1 (54.2% F, mean age 13.7y) and Cohort 2 (54.9% F, mean age 14.5y)  *Cohort 1 had higher ‘depressogenic’ risk; Cohort 2 significantly older  *N=1,733 for 12 month follow-up, 207 (12%) met criteria for clinical depression | | ↑ cortisol awakening response:  OR=7.1, 95% CI: 4.3-11.8, p<0.0001 in classes with high levels of CAR and depressive symptoms from both cohorts | |  |
|  |  |  |  |  | |  | |  |
|  |  | Raison *et al*, 2009 | CSF CRH | N=24 HCV-positive subjects (37.5% F, no current depressive disorder diagnosis for 6 months; no psychotropic drugs for 4 weeks, no fluoxetine for 8 weeks) that were treated with (N=12, 25% F, mean age 48.3y) or without (N=12, 50% F, mean age 48.3y) IFN-α for 12 weeks  *IFN-α treated subjects exhibited significantly higher MADRS scores than controls | | No significant difference of CSF CRH levels between groups | |  |
|  |  |  |  |  | |  | |  |
|  |  | Salomon *et al*., 2003 | CSF orexin-A  *sampled under constant bed rest | N=29 (15 depressed – 66.7% F, mean age 39.0y)  *3 with bipolar type I, 4 bipolar type II, 1 first episode, rest are unipolar depressives | | ↑ basal orexin levels  ↓ orexin levels after sertraline treatment  *Significantly less diurnal variation of orexin levels compared to HC | |  |
|  | |  |  |  | |  | |  |
|  | | Vreeburg *et al.,* 2009 | Salivary Cortisol | N=1,588 (579 remitted depression [70.5% F, mean age 44.7y], 701 MDD [65.3% F, mean age 42y], 308 HC [56.8% F, mean age 47.8y]) | | ↑ cortisol awakening response:  current MDD has higher overall cortisol levels vs. HC (p<0.001) but course of CAR over time similar to HC (p=0.46); remitted MDD has higher CAR (p=0.007) and different CAR time course (p=0.008) vs. HC; remitted and current MDD have similar overall CAR (p=0.25) and time course (p=0.15) | |  |
|  | |  |  |  | |  | |  |
|  | |  |  |  | |  | |  |
| Thyroid markers | | Berlin *et al*., 1999 | Serum TSH | N = 94 with DSM-III-R criteria for Major Depression [mean age 44y, 70% F] with 68% having recurrent depression and 64% with melancholia | | Subjects with TSH ≥ upper 25^th^  percentile of normal range were  more likely to have recurrent  depression, higher number of MDEs,  higher number of previous suicide  attempts compared to < 25^th^ percentile  (age-adjusted p<0.05) | |  |
|  | |  |  |  | |  | |  |
|  | | Brouwer *et al.*, 2005 | Serum TSH, free thyroxine, tri-iodothyronine, serum cortisol, TPO antibody, 24-h urinary excretion of cortisol | N = 113 Major Depressed patients [mean age 47, 70% F] with 28% having melancholic symptoms, 22% with atypical symptoms, 44% having current episode, 52% with recurrent depression | | TSH slightly higher in depressed  subjects compared to control  (p<0.001) independent of subclinical  hypothyroid status and/or presence of  TPO antibodies. HPT axis parameters  similar in both groups. Serum cortisol  lower in atypical depression compared  to non-atypical depression (p=0.01) | |  |
|  | |  |  |  | |  | |  |
|  | | Chueire *et al*., 2007 | Serum TSH, free T4, TPO antibody | Total N=323  Group I with elevated TSH: N=252 (73% F, median age 67y)  Group II diagnosed with depression: N=71 (63% F, median age 67y) | | Group I: 58.3% of subclinical hypo-  thyroid patients observed to have  depression.  Group II: 30.9% had elevated TSH,  depression observed more in  subclinical hypothyroidism vs. overt  hypothyroidism (p<0.001).  Increased risk of depression in sub-  clinical hypothyroidism (OR=4.886;  95% CI = 2.768-8.627) | |  |
|  | | Demet *et al*., 2002 | TSH, T3, T4 | N=62; 32 hyperthyroidism subjects and 30 euthyroid subjects  Hyperthyroid: median age 47.2y, 68.8% F  Euthyroid: median age 41.3y, 90% F  *authors report no significant difference between gender and age between both groups | | Depression and anxiety scores  significantly greater in hyper-  thyroidism group (p<0.05). | |  |
|  | |  |  |  | |  | |  |
|  | | Holtmann *et al*., 2010 | Serum TSH, free T3, free T4 | N=53 with high CBCL-DP scores (mean age 11.2y, 45.3% F) vs. 61 controls (mean age 13y, 50.8% F) | | TSH significantly higher in  group with high CBCL-DP score  (p=0.04). No significant difference  in free T3 and T4 levels between  groups. | |  |
|  | |  |  |  | |  | |  |
|  | | Blum *et al*., 2015 | Serum TSH, free T4 | N=606 (41% F, mean age 75y), antidepressant naïve | | Prospective study to determine  association of subclinical thyroid  dysfunction and depression.  Baseline GDS scores did not differ  between subclinical hypothyroidism  and hyperthyroidism groups and  euthyroid subjects. After 3 years,  only subclinical hyperthyroidism  group had significant increase in  GDS score (1.13 [0.32-1.93]  p=0.04) compared to euthyroid  group. | |  |
|  | |  |  |  | |  | |  |
|  | | Engum *et al*., 2005 | TPO antibody | N=30,175 (age ranged 40-84y, did not specify gender %) | | Epidemiologic study. No  association between thyroid auto-  immunity and depression or anxiety. | |  |
|  | |  |  |  | |  | |  |
|  | | Park *et al*., 2010 | Serum TSH, free T4 | N=918 (497 age 65-74, 17.7% SCH, 55.1% F; 138 age 75-84, 19.6% SCH, 66.7% F; 283 ≥85 years old, 17.3% SCH, 53.7% F) | | GDS (Korea) scores did not differ  by presence of SCH (p>0.1). No  correlation between GDS scores,  TSH, or free T4 levels. | |  |
|  | |  |  |  | |  | |  |
|  | | Carta *et al*., 2004 | TPO antibody | N=222, obtained by randomization, stratified by age and sex (age > 18y, 57.2% F) | | Significant association between  MDD and anti-TPO+ (OR=2.7, CI  95% 1.1-6.7) | |  |
|  | |  |  |  | |  | |  |
|  | | Fam *et al*., 2015 | TSH receptor antibody concentrations | N=47 F, adult, non-psychotic current MDD subjects compared to 80 F controls | | Higher TSH receptor antibody  concentration in depressed  group compared to controls  (p<0.001). Area under ROC curve  was 0.80 (95% CI, 0.73-0.88).  Higher TSH receptor antibody  concentration associated with  greater depression severity scores  (r=0.33, p<0.05).  Thyroid function, other thyroid  antibodies not associated with  depression severity | |  |
|  | |  |  |  | |  | |  |
|  | | Müssig *et al*., 2012 | TPO antibody | Age range 18-60y, mean age 64y, 88% F, diagnosed with Hashimoto’s thyroiditis, but euthyroid | | Higher TPO antibody  concentrations associated with  poor physical and psychiatric  well-being (p<0.05), and poorer  results in indices of somatization  obsessive-compulsive symptoms,  and depression (p<0.02) | |  |
|  | |  |  |  | |  | |  |
|  | | van de Ven *et al*., 2012 | Serum TSH, free T4, TPO antibody | N=1125, mean age 56.8y, 48.5% F | | No association between TSH,  free T4 concentrations and BDI  scores, current depression,  lifetime depression diagnosis.  Increased risk of life-time depression  diagnosis with + TPO antibody  (RR=1.4, 95% CI 1.0-2.1; p=0.04) | |  |
|  | |  |  |  | |  | |  |
|  | | Wei *et al*., 2014 | Hair T3 and T4 | N=60 (30 with MDD vs. 30 controls), age 18-55y, 100% F | | T3 and T4 levels were significantly  lower than controls (p<0.001).  T3 and T4 levels did not correlate  with HAMD and HAMA scale  scores. | |  |
|  | |  |  |  | |  | |  |
|  | |  |  |  | |  | |  |
| Inflammatory  Markers | | Bremmer *et al*., 2008 | Plasma CRP, IL-6 | N=1285 age 65y and older (1094 with no depression [47.3% F, mean age 75.2y]; 153 with sub-threshold depression [71.9% F, mean age 76.8y]; 38 with MDD [73.7% F, mean age 75.9y])  *All depressed had CES-D score of ≥ 16  *Note – 805 (51.7%) of initial subjects lost to attrition | | 1.) Higher CES-D scores in subjects with depressive symptoms had elevated CRP (p=0.018), although lost significance when controlling for confounding variables (β=0.24, p=0.381); No significant difference in CRP between all groups  2.) High IL-6 levels not significantly associated with higher CES-D scores (p=0.316)  3.) Odds Ratios  **High IL-6** (≥5pg/mL) ORs in:  Sub-threshold depression – OR=0.57 (95% CI: 0.29-1.12)  MDD – OR=2.49 (95% CI: 1.07-5.80)  *No differences between recurrent or 1^st^ episode groups  *Fully adjusted MDD OR=3.18 (95% CI: 1.08-9.34) in N=1081 respondents with CRP levels <10mg/mL  **High CRP** (≥3.2mg/L) ORs in:  Sub-threshold depression – OR=1.08 (95% CI: 0.73-1.60)  MDD – OR=1.26 (95% CIL 0.59-2.67)  *Plasma levels of CRP and IL-6 correlated (r=0.22, p<0.001) | |  |
|  |  | Frodl *et al.,* 2012 | Plasma IL-6, CRP | N=83 (40 MDD - 60% F, mean age 41.4y) | | ↑ IL-6 negatively affects left (p=0.044) and right (p=0.046) hippocampal volumes;  ↑ IL-6 (Z=2.1, p=0.04) and ↑ CRP (Z=2.9, p=0.004) associated with MDD compared to HC | |  |
|  |  |  |  |  | |  | |  |
|  |  | Mikova *et al*., 2001 | Serum IL-8, TNF-α, CC-16, IL-6, IL-2 receptor | N=53 (28 MDD [82.1% F, mean age 47.3y]: 19 first episode, 9 recurrent; 10 with MS [50% F, mean age 43.7y]; 15 HC [53.3% F, mean age 42y]) | | ↑ IL-8 in MDD than to MS and HC (MDD>HC>MS)  ↑ TNF-α in MDD and MS than to HC (MDD>MS>HC)  ↑ CC16 in MS than in MDD or HC (MS>MDD>HC)  *No significant difference of IL-6 (only trend of higher IL-6 in MDD) or IL-2 receptor concentrations between MDD, MS, and HC groups | |  |
|  |  |  |  |  | |  | |  |
|  |  | Motivala *et al*., 2005 | Serum IL-6, sICAM, MCP-1, IL-6sR | N=40 M subjects (22 non-medicated, current MDD with mean age 44.4y)  *depressed had current smokers while HC did not | | ↑ IL-6 (F=6.75, p=0.01) and ↑ sICAM (F=5.97, p=0.02)  *MCP-1 and IL-6sR did not significantly differ from HC  *Total HDRS scores correlated with IL-6 (r=0.40, p=0.01) and sICAM (r=0.36, p=0.02) although depressive symptom severity independent of subjective sleep disturbance did not correlate with any inflammatory markers | |  |
|  |  |  |  |  | |  | |  |
|  | | Raison *et al*., 2009 | CSF IFN-α, IL-6, soluble receptors sTNF-R2 and IL-6SR, MCP-1  Plasma IFN-α, MCP-1, IL-1β, TNF-α | N=24 HCV-positive subjects (37.5% F, no current depressive disorder diagnosis for 6 months; no psychotropic drugs for 4 weeks, no fluoxetine for 8 weeks) that were treated with (N=12, 25% F, mean age 48.3y) or without (N=12, 50% F, mean age 48.3y) IFN-α for 12 weeks  *IFN-α treated subjects exhibited significantly higher MADRS scores than controls | | CSF changes in IFN-α treated group:  ↑ IFN-α (p=0.0001), IL-6 (p=0.005), MCP-1 (p=0.001), sIL-6R (p=0.03)  *Only trend ↑ sTNF-R2 between the two groups  Plasma changes in IFN-α treated group:  ↑ IFN-α (p=0.0001), MCP-1(0.005),  *No significant difference of IL-1β, IL-6, TNF-α levels between groups  *Trend ↑ of sTNF-R2  Correlations between Plasma and CSF measures  Plasma lnIFN-α correlated with CSF IFN-α (r^2^=0.26, p<0.011) and CSFlnIL-6 (r^2^=0.20, p<0.03) although not significant after controlling for variables; All other correlations also did not reach statistical significance | |  |
|  | |  |  |  | |  | |  |
|  | | Rothermundt *et al*., 2001 | Absolute monocyte count; whole blood IL-1β; serum α_2_-macroglobulin, CRP, haptoglobin | N=86 (43 MDD subjects - 65.1% F, mean age 44.45y; 18 with single episode, 12 with moderate severity, 6 with severe severity; 25 rDD with 6 with moderate severity, 19 with severe severity; 22 MDD subjects with melancholic features) | | ↑ absolute monocyte count in patients with **non-melancholic** depression as compared to either MDD with melancholic features or HC (p<0.05)  ↑ α_2_-macroglobulin in **non-melancholic** MDD (p<0.05) compared to those that do and HC groups  *No significant difference of IL-1β levels among HC and both MDD subgroups  *Haptoglobin levels unchanged in both MDD subgroups | |  |
|  | |  |  |  | |  | |  |
|  | | Szuster-Ciesielska *et al*., 2008 | Serum IL-6, TNF-α | N=59 (29 depressive disorder subjects divided into 23 unipolar and 6 bipolar: 51.7% F, mean age 48.2y)  *All were smokers, no medications for 3-7 days | | ↑ IL-6 (p<0.05); no significant difference of TNF-α levels compared to HC | |  |
|  | |  |  |  | |  | |  |
|  | | Uddin *et al*., 2011 | Serum CRP, IL-6 | N=100 (33 with history of lifetime depression – 69.7% F, mean age 43.5y) | | ↑ CRP (p<0.01)  Trend of ↑ IL-6 (p=0.06) | |  |
|  | |  |  |  | |  | |  |
|  | |  |  |  | |  | |  |
| Oxidative Stress Markers | | Bilici *et al*., 2001 | Plasma and Erythrocyte CAT, GPX, GSH-R, MDA, SOD | N=62 (30 recurrent MDD;12 with melancholic features [66.7% F, mean age 40.4y, mean 3.8 episodes] and 18 without [72.2% F, mean age 42.2y, mean 3.2 episodes]; all are medication-naïve for 2 months) | | MDD with melancholic:  ↑ GSH-R, MDA in plasma;  ↑ GPX, SOD, MDA in erythrocytes  MDD without melancholic:  ↑ MDA in plasma;  ↑ SOD, MDA in erythrocytes  *All results statistically significant with p<0.05 (ANOVA)  Plasma GSH-R, MDA levels and erythrocyte GPX, SOD levels **discriminate between MDD and HC** with only erythrocyte SOD discriminatory among all groups (for MDD subjects – sensitivity 52.5% and specificity 90.6%; 100% sensitivity for MDD with melancholic compared to 50% for MDD without melancholic) | |  |
|  |  |  |  |  | |  | |  |
|  |  | Chung *et al*., 2013 | Urinary F2 isoprostane (2,3-dinor-5,6-dihydro-15-F2-Isoprostane) | N=54 (18 MDE, free of psychotropics for 1 month or 2 months free of fluoxetine [67% F, mean age of 32.2y])  *Subgroup was treated with either sertraline or bupropion for 8 weeks | | ↑ F2 isoprostane excretion (p=0.003)  *At baseline when depressive severity was high, no significant correlation of urinary F2 isoprostanes with mood or depressive symptoms  F2 isoprostane excretion **increased** after treatment with sertraline or bupropion (p=0.04)  *Increase in F2 isoprostane excretion after treatment correlated with decrease in HAM-D scores significantly (rho=-0.76, p=0.02) | |  |
|  |  |  |  |  | |  | |  |
|  |  | Forlenza & Miller, 2006 | Serum 8-OHdG | N=169 (84 MDD [81% F, mean age 28.7y] medication-naïve subjects divided into major [N=62] and minor [N=22] depression)  *N=59 with rDD; N=25 with single episode  *More smokers, less education in depressed group | | ↑ 8-OHdG levels compared to HC (F[1,153]=4.83, p=0.029)  *Major depressed subjects had significantly higher 8-OHdG levels compared to HC (F[1,141]=7.81, p=0.006); Minor depressed marginally higher 8-OHdG levels compared to HC (F[1,80]=3.54, p=0.062)  *rDD associated with higher levels of oxidative damage compared to other depressed and no depression | |  |
|  |  |  |  |  | |  | |  |
|  |  | Khanzode *et al*., 2003 | Serum MDA, SOD; plasma ascorbic acid | N=102 (62 MDD [32 in fluoxetine group and 30 in citalopram group] with 54.8% F, mean age 40.85y) | | ↑ MDA and ↑ SOD (p<0.001 for both) and ↓ ascorbic acid (p<0.01); positive correlation between MDA and SOD levels (r=0.37, p<0.05) | |  |
|  |  |  |  |  | |  | |  |
|  |  | Kodydková *et al*., 2009 | Whole blood hemolyzed erythrocyte CAT, GSH-R, GPX1, SOD activity; serum PON1, CD activity | N=70 elderly (mean age 65y) F (35 with depressive disorder, medication-naïve) | | ↑ activity of GSH-R (p<0.05) and SOD (p<0.001)  ↑ CD (p<0.05)  ↓ activity of GPX1  *Activity of GPX1 positively correlate with GSH levels (r=0.284, p<0.05)  *No difference of activities of CAT or PON1 compared to HC | |  |
|  |  |  |  |  | |  | |  |
|  |  | Lapidus *et al.,* 2014 | ^1^H MRS GSH | N=21 (11 MDD – 54% F, mean age 34.6y) | | ↓ GSH correlates with severity of anhedonia in MDD (p=0.01); GSH has no significant correlation with depression severity (p>0.1) | |  |
|  |  |  |  |  | |  | |  |
|  |  | Pomara *et al*., 2012 | CSF F2-isoprostanes | N=47 (28 MDD - 36% F, mean age 66.5y) of cognitively intact elderly  *21 of MDD population had recurrent episodes  *HC had 63% F | | ↑ CSF isoprostane levels (p=0.001, Cohen’s d=0.89)  *Inverse correlation between Aβ42 and F2-isoprostane levels (r=-0.331, p=0.04) | |  |
|  |  |  |  |  | |  | |  |
|  |  | Sarandol *et al*., 2007 | Plasma MDA, GPX, RBC SOD activity, TAOC | N=141 (87 MDD - 82.8% F, mean age 40y, medication free for 3 weeks)  *MDD divided into mild (HDRS 15-18, N=36), moderate (HDRS 19-22, N=40), and severe (HDRS ≥ 21, N=20) subgroups | | ↑ MDA levels, ↑RBC SOD activity, ↓ GPX, ↓ TAOC (p<0.05)  *Significant positive correlation between severity of MDD and RBC SOD activity (r=0.419, p<0.05)  *RBC SOD activity greater in severe subgroup compared to other subgroups  *No difference between mild, moderate, and severe MDD subgroups concerning MDA levels and susceptibility of RBCs to oxidation | |  |
|  |  |  |  |  | |  | |  |
|  | | Szuster-Ciesielska *et al*., 2008 | Hydrogen peroxide production; Serum CAT, PER, SOD | N=59 (29 depressive disorder subjects divided into 23 unipolar and 6 bipolar; 51.7% F, mean age 48.2y)  *All were smokers, no medications for 3-7 days | | ↑ hydrogen peroxide release,  ↑ serum CAT, PER, SOD levels  *All p<0.05 | |  |
|  | |  |  |  | |  | |  |
|  | |  |  |  | |  | |  |
| Neurotrophins and Neurotrophic factors | | Bus *et al.,* 2015 | Serum BDNF | N=1751 (153 incident depression [75.2%, mean age 44.1y], 420 remitted depression [64.3% F, mean age 42.9y], 310 persistent depression [65.2% F, mean age 44.9y]) with 868 HC | | ↓ in remitted depression (p=0.011);  ↓↓↓ in persistent depression (p=0.001); | |  |
|  |  |  |  |  | |  | |  |
|  |  | de Azevedo Cardoso  *et al.,* 2014 | Serum BDNF, NGF, GDNF | N=240 young subjects (120 MDD, 120 HC) between ages of 18-29 years old | | ↓ BDNF in all MDD patients (p≤0.001); ↓ BDNF in long-term depressed women (p=0.005) but no association in men (p=0.583)  ↑ NGF in all MDD patients (p≤0.001); ↑ NGF in depressed women with longer durations (p=0.005) and higher anxiety (p=0.011) but ↓ NGF in depressed men (p=0.026) and trend ↓ NGF in men with higher suicidal risk (p=0.080)  ↓ GDNF in all MDD patients (p≤0.001); no gender effects | |  |
|  | |  |  |  | |  | |  |
|  | | Deveci *et al*., 2007 | Serum BDNF | N=65 (24 MDD antidepressant-free for 1 year, 70.8% F, mean age 33.9y); conversion disorder group (N=15, 86.7% F, mean age 30.4y); HC group (N=26, 73.1% F, mean age 32.6y) | | ↓ BDNF in MDD (F=9.702, p=0.008)  *BDNF levels in MDD not significantly different from conversion disorder group | |  |
|  | |  |  |  | |  | |  |
|  | | Diniz *et al*., 2010 | Serum BDNF | N= 71 elderly subjects (29 current MDD [1^st^ episode or recurrent] antidepressant/psychotropic-free for one month, divided into 2 groups: 14 early onset cases [78.6% F, mean age 70.0y] and 15 late onset cases [80% F, mean age 72.0y]) | | ↓ BDNF (p=0.034); BDNF levels: HC > early onset > late onset (p < 0.03)  *early onset and HC did not have significantly different BDNF levels | |  |
|  | |  |  |  | |  | |  |
|  | | Diniz *et al*., 2013 | Serum NGF | N=77 elderly subjects mean age ≥60y (38 current MDE [74% F, mean age 70.0y]; 17 previous MDE [83% F, mean age 67.0y]; 22 HC [74% F, mean age 69.0y])  *Current MDE had significantly lower MMSE scores | | ↓ NGF (p=0.002) in both depressed groups vs. HC  *No difference between previous MDE and current MDE | |  |
|  | |  |  |  | |  | |  |
|  | | Karlović *et al*., 2013 | Serum BDNF | N=264 (142 1^st^ episode MDD – 54.1% F, mean age 46.5y) | | ↓ BDNF (p<0.01)  ROC Analysis –AUC=0.892 (95% CI: 0.826-0.939, p<0.01)  If borderline BDNF value is 48.1ng/ml:  Sensitivity – 83.9%  Specificity – 93% | |  |
|  | |  |  |  | |  | |  |
|  | | Lee & Kim, 2008 | Plasma BDNF | N=82 (32 MDD psychotropic medication-free for 2 weeks - 65.6% F, mean age 44.2y)  *8 MDD subjects were TRD (62.5% F, mean age 36.6y) and 24 responders (66.7% F, mean age 46.6y) | | Baseline  Trend ↓ BDNF (p=0.33); TRD BDNF levels not significantly lower than HC (p=0.20)  After 6 weeks antidepressant  ↑ BDNF in MDD (p=0.01) with highest increase in responder group (p<0.01)  *No significant change in TRD group (p=0.83); significant difference between responder group and TRD group BDNF levels (p=0.08) | |  |
|  | |  |  |  | |  | |  |
|  | | Lee & Kim, 2009 | Platelet BDNF | N=60 (40 MDD divided into 20 suicidal [mean age 36.4y] and 20ƒ non-suicidal [mean age 45.3y]); HC mean age 34.0y  *75% F in all groups  *Suicidal MDD had earlier age of onset  *Non-suicidal MDD older than suicidal and HC | | ↓ platelet BDNF (p<0.01) in both MDD groups  *PRP BDNF significantly lower than HC; PPP results were not significant  *No differences between suicidal and non-suicidal groups  *Recurrent MDD patients had significantly lower mean platelet BDNF than 1^st^ episode patients (Z=-2.127, p=0.033) | |  |
|  | |  |  |  | |  | |  |
|  | | Oral *et al*., 2012 | Serum BDNF | N=79 (39 MDD, 71.8% F, mean age 26.3y) | | ↓ BDNF (p<0.001); recurrent MDD had lower mean BDNF levels than 1^st^ episode MDD (p<0.001) | |  |
|  | |  |  |  | |  | |  |
|  | | Martino *et al*., 2013 | Serum NGF | N=62 (30 MDD, 80% F, mean age 48.27y) | | ↓ NGF at baseline (p=0.002); further ↓ NGF levels after duloxetine treatment | |  |
|  | |  |  |  | |  | |  |
|  | | Satomura *et al*., 2011 | Serum BDNF | N=272 (109 current MDD - 58.7% F, mean age 54.4y, on antidepressants) | | ↓ BDNF (p<0.001)  Negative correlation with HAM-D scores (r=-0.19, p=0.044) | |  |
|  | |  |  |  | |  | |  |
|  | | Shimizu *et al*., 2003 | Serum BDNF | N=83 (33 MDD divided into two groups:  1.) Antidepressant-naïve MDD - N=16, 25% F, mean age 40.8y  2.) Treated MDD - N=17, 47.1% F, mean age 49.7y  *Treated MDD had significantly higher number of depressive episodes (p=0.0014) and medication-naïve MDD had significantly higher HAM-D scores (p=0.024)) | | ↓ BDNF in antidepressant-naïve compared to treated MDD (p=-.001) and HC (p=0.002) groups  *No significant difference of BDNF levels between treated MDD and HC | |  |
|  | |  |  |  | |  | |  |
|  | |  |  |  | |  | |  |
| Genomics and  Genetics | | Ancelin *et al.,* 2013 | ACE polymorphisms and genotypes from buccal DNA samples | N=1005 ≥ 65y (255 with incident clinical depression or CES-D ≥ 16 [72.94% F; 36.47% 65-69y, 35.29% 70-74y, 28.24% 75y+]  *Non-depressed (N=750, similar ages to depressed, 55.07% F) | | ↑ risk of prevalent depression associated with homozygous polymorphisms:  **rs1800764** **genotype CC** (OR=0.53; 95% CI: 0.34-0.81, p=0.004)  ***rs4291** **genotype TT** (OR=0.44; 95% CI: 0.27-0.71, p=0.001); **genotype AT** HR=0.66 (95% CI:0.46-0.93, p=0.02)  ***rs4295** **genotype GG** (OR=0.45; 95% CI: 0.28-0.72, p=0.001), **genotype CG** HR=0.67 (95% CI: 0.48-0.95, p=0.03)  **rs4311 genotype TT** (OR=0.47; 95% CI: 0.30-0.72, p=0.001)  **rs4333** **genotype TT** (OR=0.54; 95% CI: 0.35-0.83, p=0.006)  **rs4343** **genotype GG** (OR=0.54; 95% CI: 0.35-0.84, p=0.006)  **rs4351 genotype GG** (OR=0.53; 95% CI: 0.34-0.81, p=0.003)  ***Heterozygous rs4291 and rs4295** associated with **↓ risk** for incident DEP during follow-up | |  |
|  |  |  |  |  | |  | |  |
|  |  | Anttila *et al*., 2007 | 5-HT1A & BDNF (Val66Met polymorphism) genotypes from peripheral blood leukocytes | N=511 (119 TRD-MDD, 54.6% F, mean age 57.7y)  *All subjects Caucasian | | Combination of 5-HT1A GG genotype and BDNF G196A GA + AA genotypes have OR=3.178 (95% CI: 1.315-7.680, p=0.007)  *5-HT1A C1019G polymorphism alone not associated with MDD | |  |
|  |  |  |  |  | |  | |  |
|  |  | Belzeaux *et al.,* 2012 | miRNA, mRNA  *From PBMCs | N=29 (16 severe MDE – 56.25% F, mean age 54y) | | At baseline: Following transcripts were significantly dysregulated:  ↑ hsa-miR-107 (p=0.022),  ↑ hsa-miR-133a (p=0.035),  ↑ hsa-miR-148a (p=0.0279),  ↑ hsa-miR-579 (p=0.0434),  ↑ **hsa-miR-589** (p=0.00299),  ↑ hsa-miR-652 (p=0.0435),  ↑ **hsa-miR-941** (p=0.0133);  ↓ hsa-miR-200c (p=0.035),  ↓ hsa-miR-381  (p=0.035),  ↓ hsa-miR-517b (p=0.0101),  ↓ hsa-miR-636 (p=0.00567),  ↓ hsa-miR-1243 (p=0.0279);  *Only hsa-miR-589 and hsa-miR-941 demonstrated continuous overexpression throughout 8 week follow up  ↑ expression of mRNA PPT1 predicts treatment response (p=0.00005) | |  |
|  |  |  |  |  | |  | |  |
|  |  | Bocchio-Chiavetto  *et al.,* 2013 | miRNA | N=10 MDD (50% F, mean age 42.6y)  *Caucacian ethnicity | | miRNA profile after 12 weeks of escitalopram treatment: ↑ hsa-miR-130b (p=0.0027796),  ↑ hsa-miR-505 (p=0.0027796),  ↑ hsa-miR-29b-2 (p=0.0196062),  ↑ hsa-miR-26b (p=0.0200978),  ↑ hsa-miR-22 (p=0.0200978),  ↑ hsa-miR-26a (p=0.0272020),  ↑ hsa-miR-664 (p=0.0284441),  ↑ hsa-miR-494 (p=0.0299071),  ↑ hsa-let-7d (p=0.0344815),  ↑ hsa-let-7g (p=0.0344815),  ↑ hsa-let-7e (p=0.0344815,  ↑ hsa-let-7f (p=0.0344815),  ↑ hsa-miR-629 (p=0.0410412),  ↑ hsa-miR-106b (p=0.0455580),  ↑ hsa-miR-103 (p=0.0455580),  ↑ hsa-miR-191 (p=0.0455580),  ↑ hsa-miR-128 (p=0.0455580),  ↑ hsa-miR-502-3p (p=0.0455580),  ↑ hsa-miR-374b (p=0.0486532),  ↑ hsa-miR-132 (p=0.0486532),  ↑ hsa-miR-30d (p=0.0486532),  ↑ hsa-miR-500 (p=0.0486532),  ↑ hsa-miR-589 (p=0.0486532),  ↑ hsa-miR-183 (p=0.0486532),  ↑ hsa-miR-574-3p (p=0.0486532),  ↑ hsa-miR-140-3p (p=0.0486532),  ↑ hsa-miR-335 (p=0.0486532),  ↑ hsa-miR-361-5p (p=0.0486532) ↓ hsa-miR-34c-5p (p=0.0344815),  ↓ hsa-miR-770-5p (p=0.0486532) | |  |
|  |  |  |  |  | |  | |  |
|  |  | Frodl *et al.,* 2012 | GILZ mRNA | N=83 (40 MDD - 60% F, mean age 41.4y) | | ↓ GILZ expression associated with decreased hippocampal volumes in MDD (Compared to a.) HC with low GILZ mRNA: M=-0.47, p=0.003, Sidak corrected; b.) HC with high GILZ mRNA: M=-0.41, p=0.007, Sidak corrected) | |  |
|  |  |  |  |  | |  | |  |
|  |  | Fuchikami *et al*., 2011 | BDNF DNA methylation profile | N=38 (20 MDD – 60% F, mean age 45.6y)  *All Japanese | | DNA methylation pattern of CpG I BDNF gene differentiate MDD subjects from HC group (29/35 CpG units in BDNF gene significantly different) | |  |
|  |  |  |  |  | |  | |  |
|  |  | He *et al.,* 2012 | DGCR8 and AGO1 polymorphisms and genotypes | N=566 (314 MDD – 68.8% F (M – mean age 42.34y; F – mean age 47.93y)  *Significant differences between genders (F are older, have later onset, more chronic) | | Variant genotypes associated with increased depression risk:  **DGCR8 rs3757** (OR=0.71; 95% CI=0.52-0.97, p<0.03);  **AGO1 rs636832** (OR=1.35; 95% CI=1.04-1.75, p=0.02) | |  |
|  |  |  |  |  | |  | |  |
|  |  | Hwang *et al*., 2006 | Val66Met polymorphism (BDNF gene) | N= 281 (110 MDD subjects - 42.7% F, mean age 75.0y) all ≥ 60 years old | | Val66Met genotype (p=0.003) and allele frequency (p=0.001) significantly different from HC; ↑ Met/Met genotype and ↑ Met allele in geriatric depressed patients  Met/Met genotype indicated increased risk for late-life depression with OR=2.49 (95% CI: 1.40-4.46) | |  |
|  |  |  |  |  | |  | |  |
|  |  | Licinio *et al*., 2009 | BDNF SNPs | N=536 Mexican-Americans (272 MDD – 65.8% F, mean age 37.8y) | | 6 BDNF SNPs significantly associated with depression:  **rs41282918** (OR=2.13, 95% CI: 1.18-3.86, p=0.01)  **rs11030103** (OR=1.80, 95% CI: 1.18-2.74, p=0.008)  **rs12273539** (OR=1.75, 95% CI: 1.32-2.31, p=0.00009)  **rs6265** (OR=1.66, 95% CI: 1.14-1.41, p=0.009)  **rs28722151** (OR=1.48, 95% CI: 1.10-1.99, p=0.01)  **rs11030101** (OR=1.37, 95% CI: 1.05-1.78, p=0.02) | |  |
|  |  |  |  |  | |  | |  |
|  |  | Ribeiro *et al*., 2007 | rs6265 SNP of BDNF gene | N=615 Mexican-American (N=284 MDD – 66.1% F, mean age 39.0y) | | rs6265 associated with depression diagnosis (p=0.005) and increased risk (OR=1.7, 95% CI: 1.17-2.47, p=0.0053) if with GG genotype | |  |
|  |  |  |  |  | |  | |  |
|  |  | Szebeni *et al.,* 2014 | GPX1, SOD1, SOD2, CAT expression  *Oligodendrocyte samples from post-mortem human brain tissues | N=28 (14 MDD donors – mean age 51.00y)  *Both HC and MDD donors have only 1 F | | ↓ gene expression of SOD1 (UF: F=19.7, p<0.0005; BA10 white matter: F=21.2, p<0.0005), SOD2 (UF: F=20.5, p<0.0005; BA10 white matter: F=46.8, p<0.0005), GPX1 (UF: F=19.2, p<0.0005; BA10 white matter: F=25.4, p<0.0005), CAT (UF: F=15.4, p=0.001; BA10 white matter: F=21.3, p<0.0005) | |  |
|  |  |  |  |  | |  | |  |
|  |  | Uddin *et al*., 2011 | DNA methylation profiles | N=100 (33 with history of lifetime depression – 69.7% F, mean age 43.5y) | | Depressed subjects had significantly different methylation profiles concerning unmethylated genes (χ^2^=30.78, p<0.0001) and methylated genes (χ^2^=271.04, p<0.0001)  Higher rates of methylated genes related to brain development/neurogenesis, tryptophan metabolism, protease; lower rates of methylated genes related to lipoprotein, hydrolase activity.  *Higher methylation levels = lower expression levels | |  |
|  |  |  |  |  | |  | |  |
|  | |  |  |  | |  | |  |
| Proteomics, Metabonomics, Enzymatic activity | | Ding *et al.,* 2014 | Plasma analytes measured by GC/MS coupled with multivariate analysis | N=71 (46 chronic MDD - MDD patients divided into 23 ELS/MDD [mean age 29y] and 23 non-ELS/MDD [mean age 30y])  *Mostly Han Chinese (82-100%) | | Highest predictive feature metabolite combinations:  **MDD from HC** – valine, leucine, proline, glycemic acid, pyroglutamate, galactose, glucopyranose  **ELS/MDD from HC** – lactic acid, proline, glyceric acid, mannose, gluconate, tryptophane, stearic acid, cholesterol  **Non-ELS/MDD from HC** – 6-deoxidation mannopyrannose, palmitic acid, heptadecylic acid  **ELS/MDD from non-ELS/MDD** – oxalic acid, heptadecylic acid, stearic acid | |  |
|  |  |  |  |  | |  | |  |
|  |  | Lu *et al.,* 2014 | Plasma aspartic acid, glutamate, GABA, NO, Gly | N=57 (27 un-medicated melancholic, 1^st^ episode MDD - 48.1% F, 30-68y)  *All Han Chinese | | ↑ NO (p=0.005); ↓ aspartic acid (p=0.003), ↓ GABA (p=0.021), ↓ Gly (p<0.001)  *No difference in Glutamic acid from HC (p=0.646)  *Trend higher GABA in MDD subjects who had suicidal ideation (p=0.054) and anhedonia (p=0.076)  *↑ glutamic acid in M compared to F subjects (p=0.003) | |  |
|  |  |  |  |  | |  | |  |
|  |  | Raison *et al*., 2009 | CSF 5-HIAA, HVA, MHPG | N=24 HCV-positive subjects (37.5% F, no current depressive disorder diagnosis for 6 months; no psychotropic drugs for 4 weeks, no fluoxetine for 8 weeks) that were treated with (N=12, 25% F, mean age 48.3y) or without (N=12, 50% F, mean age 48.3y) IFN-α for 12 weeks  *IFN-α treated subjects exhibited significantly higher MADRS scores than controls | | No statistical difference between groups of CSF 5-HIAA, HVA, MHPG levels | |  |
|  |  |  |  |  | |  | |  |
|  |  | Wang *et al*., 2014 | Urinary peptides measured by MALDI-TOF MS | N=107 divided into training set (N=70; 42 MDD [54.8%F, mean age 32.74y]) and test set (N=37; 24 MDD [58.3% F, mean age 34.96y])  *All MDD are 1^st^ episode, drug-naïve | | Training Set  Peptide model distinguishes MDD from HC:  Sensitivity – 90.5%  Specificity – 92.9%  Accuracy – 91.4%  Test Set  Peptide model distinguishes MDD from HC:  Sensitivity – 91.7%  Specificity – 84.6%  Accuracy – 89.2%  *Identified 4/5 model peptides as fragments from serum albumin, protein AMBP, heparin sulfate proteoglycan, apolipoprotein A-I | |  |
|  |  |  |  |  | |  | |  |
|  |  | Zhang *et al.,* 2014 | Platelet 5-HT1A expression, 5-HT, SERT expression  PPP 5-HT, 5-HIAA | N=75 (53 un-medicated MDD – 86.8% F, mean age 47y)  *22 HC – 77.3% F, mean age 29.6y | | Platelet results: ↑ 5-HT1A expression (p<0.001); ↓ 5-HT (p<0.001),  *No significant difference in SERT expression between controls and MDD (p=0.059)  ↓ PPP 5-HT (p<0.001), 5-HIAA (p<0.001) | |  |
|  |  |  |  |  | |  | |  |
|  |  | Zheng *et al.,* 2013 | Urinary AZA, sorbitol, UA, QA, HA, Tyr, NMN as measured by GC/MS | N=260 divided into training set (82 MDD – 43.9% F, mean age 32.2y; 82 HC – 35.4% F, mean age 34.2y) and test set (44 MDD – 61.4% F, mean age 34.1y; 52 HC – 48.1% F, mean age 28.8y)  *Test set MDD significantly older than Test set HC group | | Urinary metabolite panel: ↑ AZA, sorbitol, UA; ↓ QA, HA, Tyr  Composite urinary metabolite biomarker panel (without NMN): AUC of 0.905 in training set, AUC of 0.837 in test set  Composite urinary metabolite biomarker panel (with NMN): AUC of 0.909 in training set, AUC of 0.917 in test set | |  |
|  | \|  \|  \|  \|  \|  \| \| --- \| --- \| --- \| --- \| --- \|   Abbreviations: ↑ - increase; ↓ - decrease; ↑↑↑ - steep increase; ↓↓↓ - steep decrease; ^1^H MRS – proton magnetic resonance spectroscopy; 5-HT - serotonin; 5-HIAA - 5-hydroxyindoleacetic acid; 5-HT1A - serotonin type 1A receptor; 5-HTT – see SERT; 8-OHdG – 8-hydroxy-deoxyguanosine; AGO1 - argonaute RISC catalytic component 1; ANOVA – analysis of variance; AUC – area under curve; AZA - azelaic acid; BA10 – broca’s area 10; BDI – Beck Depression Inventory; BDNF - brain derived neurotrophic factor; CAR – cortisol awakening response; CAT - catalase; CBCL-DP – Child Behavior Checklist Dysregulation Profile; CD – conjugated dienes; CES-D – Center for Epidemiologic Studies-Depression Scale; COX-2 – cyclooxygenase-2; CRH - corticotropin releasing hormone; CRP - C-reactive protein; CSF – cerebrospinal fluid; DPP-IV - dipeptidyl peptidase 4; DGCR8 - Digeorge syndrome critical region 8; ELS - early life stress; F2-isoPM - 2,3-dinor-5,6-dihydro-15-F2t-isoprostane; F – female; GABA - gamma-Aminobutyric acid; GC/MS - gas chromatography/mass spectroscopy; GDNF – Glial cell line-derived neurotrophic factor; GDS – Geriatric Depression Scale; GILZ - glucocorticoid-induced leucine zipper; Gly - Glycine; GPX - glutathione peroxidase; GSH-R – glutathione reductase; HA - hippuric acid; HAMA – Hamilton Anxiety Rating Scale; HAM-D – see HDRS; HC – healthy controls; HCV – hepatitis C virus; HDRS – Hamilton Depression Rating Scale; HR – hazard ratio; HVA – homovanillic acid; HPT – Hypothalamic-Pituitary-Thyroid; GSH - glutathione; IFN – interferon; IL - interleukin; IL-6sR – IL-6 soluble receptor; ln – log-transformed; M – male; MADRS – Montgomery-Asberg Depression Rating Scale; MALDI-TOF MS – matrix-assisted laser desorption ionization time-of-flight mass spectroscopy; MCP-1 – monocyte chemotactic protein; MDA – malondialdehyde; MDE – major depressive episode; MDD – major depressive disorder; MHPG – 3-methoxy-4-hydroxyphenylglycol; MMSE – Mini Mental Status Exam; MRI – magnetic resonance imaging; MS – multiple sclerosis; NGF - nerve growth factor; NMN - N-methylnicotinamide; NO - nitric oxide; OR – odds ratio; PBMC – peripheral blood mononuclear cells; PER – total peroxidase; PON – paraoxonase; PPP - platelet poor plasma; PPT1 - palmitoyl-protein thioesterase 1; PRP – platelet rich plasma; PTSD – post-traumatic stress disorder; QA - quinolinic acid; RBC – red blood cell; rDD – recurrent depressive disorder; REM – rapid eye movement; ROC – receiver operating characteristic; SCH – subclinical hypothyroidism; SD – Standard deviation; SERT - serotonin transporter; SDGS-K – Short Form Geriatric Depression Scale-Korean version; sICAM – soluble intercellular adhesion molecule; sIL-6R – soluble interleukin-6 receptor; SNP – single nucleotide polymorphism; SOD - superoxide dismutase; SOD1 - superoxide dismutase 1; SOD2 - mitochondrial superoxide dismutase; sPLA2-IIA – secretory phospholipase A2 type IIA; sTNF-R – soluble tumor necrosis factor receptor; TAOC – total antioxidative capacity; TLR - toll-like receptor; TNF - tumor necrosis factor; TPO – thyroid peroxidase; TRD – treatment resistant depression; TSH – thyroid stimulating hormone; Tyr - tyrosine; UA - uric acid; UD – unipolar depression; UF – uncinate fasciculus; WML – white matter lesion; y – years old * All changes refer to blood-based studies unless otherwise stated. | | | | | | | |
